# Supplementary material for: A Lightweight Browser-Based Tool for Collaborative and Blinded Image Analysis
Source: J Imaging. 2024 Jan 27;10(2):33. doi: 10.3390/jimaging10020033 (PMC10889326; doi:10.3390/jimaging10020033)
Supplement: Supplementary file 1 [file jimaging-10-00033-s001.zip › jimaging-2754553-Credits for icons used in Figure 1.pdf]

Credits for icons used in Figure 1:

A

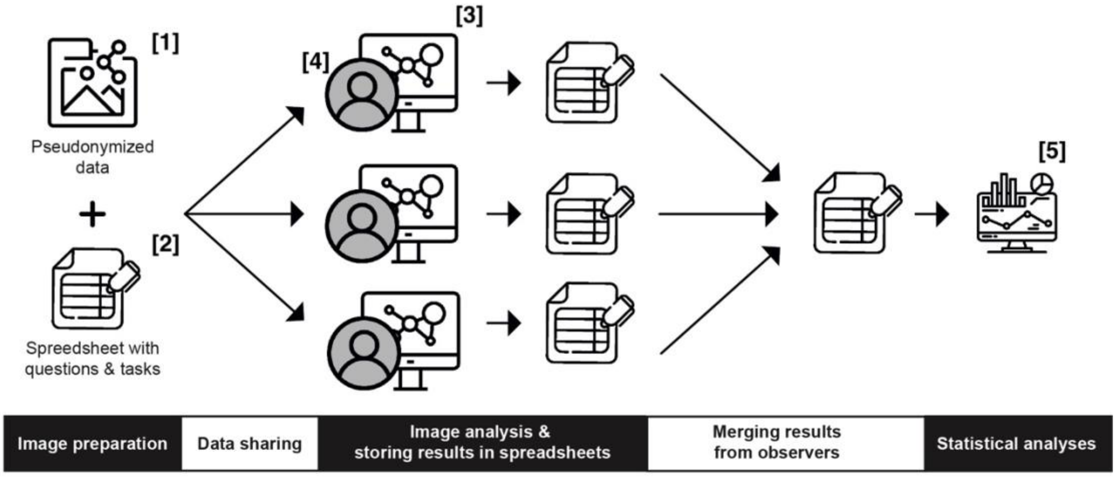

B

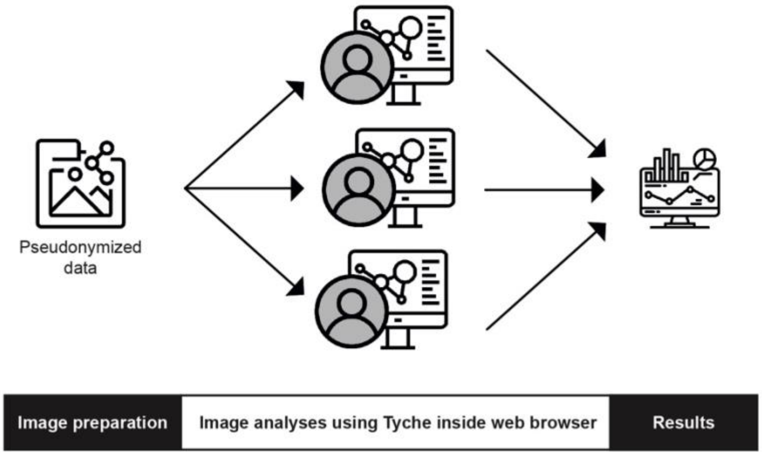

[1] designed by Smartline from Flaticon
[2] designed by Freepik from Flaticon
[3] designed by FBJan from Flaticon
[4] designed by Freepik from Flaticon
[5] designed by xnimrodx from Flaticon
