# Supplementary material for: A Lightweight Browser-Based Tool for Collaborative and Blinded Image Analysis
Source: J Imaging. 2024 Jan 27;10(2):33. doi: 10.3390/jimaging10020033 (PMC10889326; doi:10.3390/jimaging10020033)
Supplement: Supplementary file 1 [file jimaging-10-00033-s001.zip › jimaging-2754553-supplementary.pdf]

	Article title	Journal name (abbreviated)	Impact Factor	DOI	Detailed description of approach	Analyzed in random order by multiple blinded observers	Key phrase
**1**	Teriparatide ameliorates articular cartilage degradation and aberrant subchondral bone remodeling in DMM mice	J Orthop Translat	5.19	10.1016/j.jot.2022.10.015	No	Yes	*(…) evaluated by blinded observers*
**2**	ROS-responsive PPGF nanofiber membrane as a drug delivery system for long-term drug release in attenuation of osteoarthritis	NPJ Regen Med	7.02	10.1038/s41536-022-00254-3	No	Yes	*(…) and scored by 3 independent estimators who were not known the treated groups*
**3**	Vitrified Particulated Articular Cartilage for Joint Resurfacing	Am J Sports Med	6.2	10.1177/03635465221123045	No	Yes	*(...) assessed independently by 2 observers (….) blinded to the treatment (…)*
**4**	Association between radiographic hand osteoarthritis and bone microarchitecture in a population-based sample	Arthritis Res Ther	5.16	10.1186/s13075-022-02907-6	No	Yes	*(…) by 2 readers*
**5**	Synovial membrane-derived mesenchymal progenitor cells from osteoarthritic joints in dogs possess lower chondrogenic-, and higher osteogenic capacity compared to normal joints	Stem Cell Res Ther	5.12	10.1186/s13287-022-03144-z	No	Yes	*(…) randomized and scored (…) by three observers*
**6**	Modelling osteoarthritis in mice via surgical destabilization of the medial meniscus with or without a stereomicroscope	Bone Joint Res	5.85	10.1302/2046-3758.118.BJR-2021-0575.R1	No	Yes	*Supplements: (…) were scored by two experienced scorers (…)*
**7**	Theaflavin-3,3′-Digallate Protects Cartilage from Degradation by Modulating Inflammation and Antioxidant Pathways	Oxid Med Cell Longev	6.54	10.1155/2022/3047425	No	Not detailed	-
**8**	ShcA promotes chondrocyte hypertrophic commitment and osteoarthritis in mice through RunX2 nuclear translocation and YAP1 inactivation	Osteoarthritis Cartilage	6.58	10.1016/j.joca.2022.07.001	No	Yes	*Supplements: Sections were blinded and scored by three different experienced scientists*
**9**	Increased Wnt/β-catenin signaling contributes to autophagy inhibition resulting from a dietary magnesium deficiency in injury-induced osteoarthritis	Arthritis Res Ther	5.16	10.1186/s13075-022-02848-0	No	Not detailed	-
**10**	The Protective Effect of Evodiamine in Osteoarthritis: An In Vitro and In Vivo Study in Mice Model	Front Pharmacol	5.81	10.3389/fphar.2022.899108	No	Not detailed	-

**11**	Estradiol Inhibits ER Stress-Induced Apoptosis in Chondrocytes and Contributes to a Reduced Osteoarthritic Cartilage Degeneration in Female Mice	Front Cell Dev Biol	6.68	10.3389/fcell.2022.913118	No	Yes	*2 independent scientists evaluated at least three sections in order to evaluate the entire joint in a blinded manner*
**12**	Curcumin exerts chondroprotective effects against osteoarthritis by promoting AMPK/PINK1/Parkin-mediated mitophagy	Biomed Pharmacother	6.53	10.1016/j.biopha.2022.113092	No	Yes	*Each section was scored in a blinded fashion by two individuals*
**13**	Saikosaponin D Inhibited IL-1β Induced ATDC 5 Chondrocytes Apoptosis In Vitro and Delayed Articular Cartilage Degeneration in OA Model Mice In Vivo	Front Pharmacol	5.81	10.3389/fphar.2022.845959	No	Not detailed	-
**14**	Toll-like receptor 3 activation promotes joint degeneration in osteoarthritis	Cell Death Dis	8.47	10.1038/s41419-022-04680-5	No	Yes	*Two independent graders assessed (…) in a blinded manner*
**15**	Baicalein Alleviates Osteoarthritis Progression in Mice by Protecting Subchondral Bone and Suppressing Chondrocyte Apoptosis Based on Network Pharmacology	Front Pharmacol	5.81	10.3389/fphar.2021.788392	No	Yes	*Two independent experienced researchers who were blinded (...)*
**16**	Engineered adipose-derived stem cells with IGF-1-modified mRNA ameliorates osteoarthritis development	Stem Cell Res Ther	5.12	10.1186/s13287-021-02695-x	No	Yes	*(...)scored sequentially in a blinded manner by 3 observers*
**17**	Antinociceptive and chondroprotective effects of prolonged β-caryophyllene treatment in the animal model of osteoarthritis: Focus on tolerance development	Neuro-pharmacology	5.25	10.1016/j.neuropharm.2021.108908	No	No	*by an observer (...) blinded to the pharmacological treatment*
**18**	Pharmacological characterization of GLPG1972/S201086, a potent and selective small-molecule inhibitor of ADAMTS5	Osteoarthritis Cartilage	6.58	10.1016/j.joca.2021.08.012	No	No	*Supplements: (...) performed by a single observer blinded to treatment*

Supplementary Table S1: The majority of authors scored images with multiple blinded observers

The PubMed® database was screened on December 17th using the keyword “oarsi score”. Free full text articles from 2022 with an impact factor equal or greater than five were selected. Articles were screened whether they analyzed the images blinded and if multiple analyzers were included.

References

1 Li G, Liu S, Chen Y, Xu H, Qi T, Xiong A, et al. Teriparatide ameliorates articular cartilage degradation and aberrant subchondral bone remodeling in DMM mice. J Orthop Transl. 2023;38:241–55.

2 Wu J, Qin Z, Jiang X, Fang D, Lu Z, Zheng L, et al. ROS-responsive PPGF nanofiber membrane as a drug delivery system for long-term drug release in attenuation of osteoarthritis. Npj Regen Medicine. 2022b;7(1):66.

3 Wu K, Yong KW, Ead M, Sommerfeldt M, Skene-Arnold TD, Westover L, et al. Vitrified Particulated Articular Cartilage for Joint Resurfacing: A Swine Model. Am J Sports Medicine. 2022c;50(13):3671–80.

4 Ma C, Aitken D, Wu F, Squibb K, Cicuttini F, Jones G. Association between radiographic hand osteoarthritis and bone microarchitecture in a population-based sample. Arthritis Res Ther. 2022;24(1):223.

5 Teunissen M, Ahrens NS, Snel L, Narcisi R, Kamali SA, Osch GJVM van, et al. Synovial membrane-derived mesenchymal progenitor cells from osteoarthritic joints in dogs possess lower chondrogenic-, and higher osteogenic capacity compared to normal joints. Stem Cell Res Ther. 2022;13(1):457.

6 Hu W, Lin J, Wei J, Yang Y, Fu K, Zhu T, et al. Modelling osteoarthritis in mice via surgical destabilization of the medial meniscus with or without a stereomicroscope. Bone Joint Res. 2022;11(8):518–27.

7 Teng Y, Jin Z, Ren W, Lu M, Hou M, Zhou Q, et al. Theaflavin-3,3′-Digallate Protects Cartilage from Degradation by Modulating Inflammation and Antioxidant Pathways. Oxid Med Cell Longev. 2022;2022:3047425.

8 Abou-Jaoude A, Courtes M, Badique L, Mahmoud DE, Abboud C, Mlih M, et al. ShcA promotes chondrocyte hypertrophic commitment and osteoarthritis in mice through RunX2 nuclear translocation and YAP1 inactivation. Osteoarthr Cartilage. 2022;30(10):1365–75.

9 Bai R, Miao MZ, Li H, Wang Y, Hou R, He K, et al. Increased Wnt/β-catenin signaling contributes to autophagy inhibition resulting from a dietary magnesium deficiency in injury-induced osteoarthritis. Arthritis Res Ther. 2022;24(1):165.

10 Xian S, Lin Z, Zhou C, Wu X. The Protective Effect of Evodiamine in Osteoarthritis: An In Vitro and In Vivo Study in Mice Model. Front Pharmacol. 2022;13:899108.

11 Dreier R, Ising T, Ramroth M, Rellmann Y. Estradiol Inhibits ER Stress-Induced Apoptosis in Chondrocytes and Contributes to a Reduced Osteoarthritic Cartilage Degeneration in Female Mice. Frontiers Cell Dev Biology. 2022;10:913118.

12 Jin Z, Chang B, Wei Y, Yang Y, Zhang H, Liu J, et al. Curcumin exerts chondroprotective effects against osteoarthritis by promoting AMPK/PINK1/Parkin-mediated mitophagy. Biomed Pharmacother. 2022;151:113092.

13 Wu X, Zhao K, Fang X, Lu F, Cheng P, Song X, et al. Saikosaponin D Inhibited IL-1β Induced ATDC 5 Chondrocytes Apoptosis In Vitro and Delayed Articular Cartilage Degeneration in OA Model Mice In Vivo. Front Pharmacol. 2022d;13:845959.

14 Stolberg-Stolberg J, Boettcher A, Sambale M, Stuecker S, Sherwood J, Raschke M, et al. Toll-like receptor 3 activation promotes joint degeneration in osteoarthritis. Cell Death Dis. 2022;13(3):224.

15 Yi N, Mi Y, Xu X, Li N, Zeng F, Yan K, et al. Baicalein Alleviates Osteoarthritis Progression in Mice by Protecting Subchondral Bone and Suppressing Chondrocyte Apoptosis Based on Network Pharmacology. Front Pharmacol. 2022;12:788392.

16 Wu H, Peng Z, Xu Y, Sheng Z, Liu Y, Liao Y, et al. Engineered adipose-derived stem cells with IGF-1-modified mRNA ameliorates osteoarthritis development. Stem Cell Res Ther. 2022a;13(1):19.

17 Mlost J, Kac P, Kędziora M, Starowicz K. Antinociceptive and chondroprotective effects of prolonged β-caryophyllene treatment in the animal model of osteoarthritis: Focus on tolerance development. Neuropharmacology. 2022;204:108908.

18 Clement-Lacroix P, Little CB, Smith MM, Cottereaux C, Merciris D, Meurisse S, et al. Pharmacological characterization of GLPG1972/S201086, a potent and selective small-molecule inhibitor of ADAMTS5. Osteoarthr Cartilage. 2022;30(2):291–301.
